# Kinesin Eg5 Targeting Inhibitors as a New Strategy for Gastric Adenocarcinoma Treatment

**DOI:** 10.3390/molecules24213948

**Published:** 2019-10-31

**Authors:** Guya Diletta Marconi, Simone Carradori, Alessia Ricci, Paolo Guglielmi, Amelia Cataldi, Susi Zara

**Affiliations:** 1 Department of Medical, Oral and Biotechnological Sciences, University “G. d’Annunzio” of Chieti-Pescara, Via dei Vestini 31, 66100 Chieti, Italy; guya.marconi@virgilio.it; 2 Department of Pharmacy, University “G. d’Annunzio” of Chieti-Pescara, Via dei Vestini 31, 66100 Chieti, Italy; alessia.ricci@studenti.unich.it (A.R.); amelia.cataldi@unich.it (A.C.); susi.zara@unich.it (S.Z.); 3 Department of Drug Chemistry and Technologies, Sapienza University of Rome, P.le A. Moro 5, 00185 Rome, Italy; paolo.guglielmi@uniroma1.it

**Keywords:** Eg5, kinesin, AGS, thiadiazoline, monoastral spindle, K858

## Abstract

The Kinesins are proteins involved in several biological processes such as mitosis, intracellular transport, and microtubule movement. The mitotic process is allowed by the correct formation of the mitotic spindle which consists of microtubules originating from the spindle poles. In recent years, kinesin Eg5 inhibitors were studied as new chemotherapeutic drugs, due to the lack of side effects and resistance mechanisms. The aim of this work was to investigate the molecular signaling underlying the administration of novel kinesis Eg5 inhibitors in an in vitro model of gastric adenocarcinoma. Data obtained from analogues of **K858** led us to select compounds **2** and **41**, due to their lower IC_50_ values. The ability of kinesin inhibitors to induce apoptosis was investigated by evaluating Bax and Caspase-3 protein expression, evidencing that compound **41** and **K858** markedly raise Bax expression, while only compounds **2** and **41** co-administrated with **K858** trigger Caspase-3 activation. The inhibition of mitotic spindle was measured by β-tubulin immunofluorescence analysis revealing monopolar spindles formation in gastric cancer cells treated with compounds **2**, **41**, and **K858**. Nitric Oxide Synthase (NOS-2) and Matrix Metalloproteinase 9 (MMP-9) expression levels were measured finding a NOS-2-mediated downregulation of MMP-9 when compound **41** and **K858** are co-administered. However, this is in contrast to what was reported by migration assay in which both novel compounds and **K858** in monotherapy markedly reduce cell migration. This work remarks the importance of understanding and exploring the biological effects of different novel Eg5 kinesin inhibitors administered in monotherapy and in combination with **K858** as potential strategy to counteract gastric cancer.

## 1. Introduction

Kinesin superfamily proteins, also known as KIFs, are essential molecular motors that directionally transport several cargos, including membranous organelles, protein complexes, and mRNAs, and also mediate cell division and microtubule movement. They are categorized into 14 subfamilies on the basis of sequence homology and classified as mitotic kinesins, which are involved in cell division, and non-mitotic kinesins, and are principally involved in intracellular transport. The mitotic process is allowed by the correct formation of the mitotic spindle which consists of microtubule polymers containing α/β tubulin dimers which emerge from the spindle poles and tie to the condensed chromosomes by means of a specific structure, the kinetochore, at the centromere [1].

The mitotic spindle is a relevant target in cancer therapy and various drugs interfering with microtubule dynamics are approved for clinical use. Recently, mitotic kinesins represented a promising molecular target to develop new chemotherapeutic agents due to their lack of many side effects, such as neuropathy and resistance mechanisms shown by Taxanes and Vinca alkaloids, which restrict the use of these chemotherapeutics [2]. Indeed, several compounds inhibiting mitotic kinesins such as Eg5 and centromere-associated protein E (CENPE) have been inserted in phase I and II of clinical trials either as monotherapies or in combination [3,4].

Eg5 (also known as KIF11 or Kinesin Spindle Protein), a member of the kinesin-5 family, plays a central role in the formation and maintenance of the bipolar spindle during mitosis. Eg5 expression block damages the separation of duplicated centrosomes, resulting in cell-cycle arrest and triggering apoptotic cell death in tumor cells. Indeed, recent studies reported that Eg5 expression is associated with several malignancies, such as hepatic carcinoma, lung, pancreatic, gastric, colorectal, and prostate cancers [5,6]. In particular, an overexpression of kinesin Eg5 has been demonstrated in gastric cancer [7], a malignant tumor associated with high-grade mortality whose pathogenesis is still uncertain [8,9].

Based on this knowledge, starting from monastrol, the first Eg5 inhibitor discovered in 1999 [10], a number of Eg5 inhibitors were then discovered, synthetized, and used in basic and clinical research [11]. In our laboratory, seven novel kinesin Eg5 inhibitors (**2**, **4**, **26**, **30**, **31**, **41**, and **44**) were synthesized keeping constant the thiadiazoline core nucleus and modifying the C5 substituents of **K858**, the most common Eg5 inhibitor used in cancer therapies, due to their promising inhibition of the basal Eg5 ATPase activity (0.84 < IC_50_ (μM) < 7.5) in vitro [12].

The aim of this study was to investigate for the first time the biological effects and the potential mechanism of action of **K858** and novel thiadiazoline inhibitors of kinesin Eg5 in a cell model of human gastric adenocarcinoma in which cell viability, apoptosis occurrence, and invasiveness potential were evaluated.

## 2. Results

### 2.1. Effects of Novel Kinesin Eg5 Inhibitors and **K858** on AGS Cell Viability

**Figure 1:**
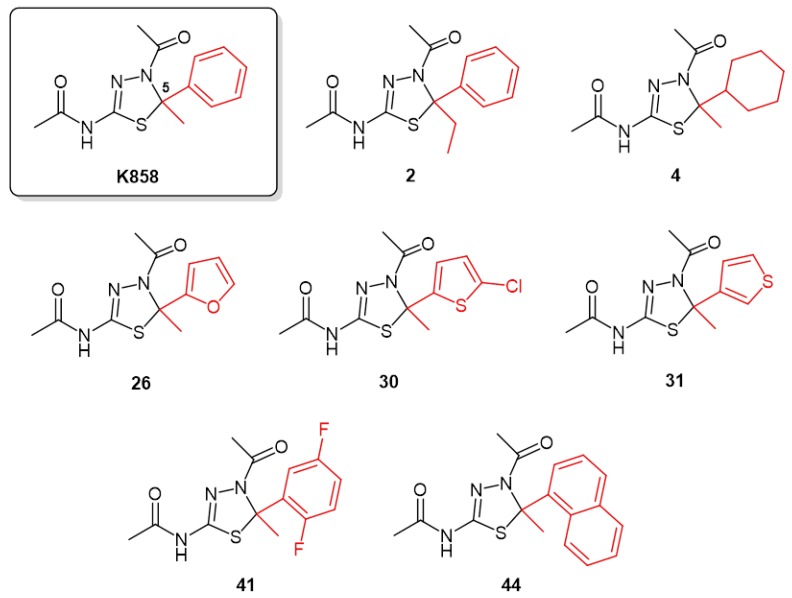
Molecular structures of selected thiadiazoline-based kinesin Eg5 inhibitors.

Potent kinesin Eg5 inhibitors, compounds **2**, **4**, **26**, **30**, **31**, **41**, and **44**, characterized by the thiadiazoline nucleus present in the structure of **K858** and endowed with different substituents at C5 (Figure 1), were enrolled in this study on the basis of the reported in vitro inhibition of the basal Eg5 ATPase activity in the range 0.84 < IC_50_ (μM) < 7.5 [12].

**Figure 2:**
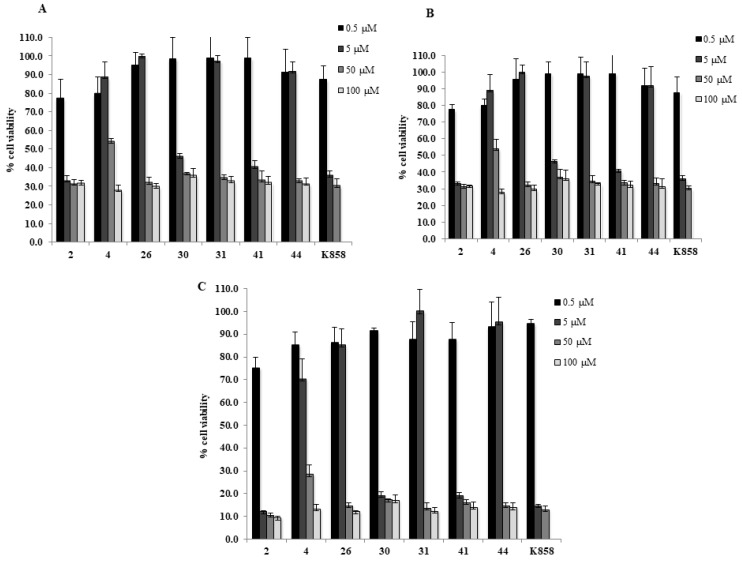
**MTT cell viability assay on AGS.** Histograms represent the viability dose-response of AGS cells exposed to different concentrations of compounds **2**, **4**, **26**, **30**, **31**, **41**, **44**, and **K858** (from 0.5 to 100 μM) for 24, 48, and 72 h (**A**–**C**, respectively). Metabolic activity was assessed using MTT assay and normalized to control cells treated with DMSO (0.2% as final concentration).

Compounds **2**, **4**, **26**, **30**, **31**, **41**, and **44** were screened as single agent at 0.5, 5, 50, and 100 μM and **K858** at 0.5, 5, and 50 μM for 24, 48, and 72 h on a human gastric adenocarcinoma AGS cell line by means of a MTT viability assay (Figure 2). Compound **4** appears well tolerated in AGS cells considering that, for doses lower than 100 µM, the recorded cell viability percentage reaches approximately 50%, especially after 24 and 48 h of treatment. Compounds **26**, **31**, and **44** show a similar trend and reveal a higher toxicity with respect to compound **4** considering that they affect cell viability at doses higher than 5 µM at all tested experimental times. When compounds **26**, **31**, and **44** are administered at lower doses (0.5 and 5 µM) the cell viability percentage is always higher than 80%. Conversely, compounds **2**, **30**, and **41** exhibit a cell viability lower than 50% starting from 5 µM at all tested experimental times.

Based on the obtained results, three novel and potent kinesin Eg5 inhibitors, compounds **2**, **30,** and **41** are identified as the most effective molecules and administered for 24 and 48 h at doses ranging from 3.125 up to 100 μM in order to identify a subtoxic dose suitable for further molecular investigations; **K858** was administered starting from 0.05 up to 50 μM, according to the literature [13,14,15].

**Figure 3:**
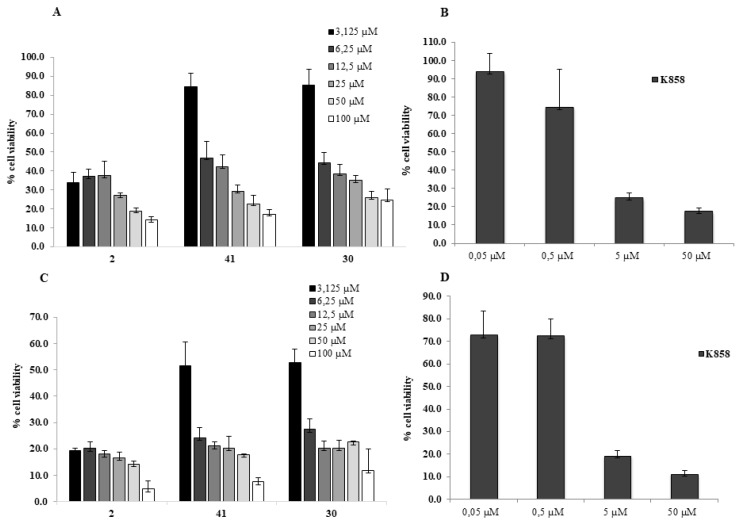
**MTT cell viability assay on AGS.** (**A**,**C**) Histograms represent the viability dose-response of AGS cells exposed to different concentrations of **2**, **41**, **30** (from 3.125 to 100 μM) for 24 and 48 h. (**B**,**D**) Histograms represent the viability dose-response of AGS cells exposed to different concentrations of **K858** (from 0.05 to 50 μM) for 24 and 48 h. Proliferation was assessed using MTT assay and normalized to control cells treated with DMSO (0.2% as final concentration).

The effects of compounds **2**, **30**, **41**, and **K858** were investigated through MTT test on human AGS cells (Figure 3). IC_50_ values measured after 24 h of treatment were about 3 μM for compound **2** and about 6 μM for both compounds **30** and **41**. Compound **2** exhibits approximately 30% of viable cells at 3.125 μM comparable to cell viability percentage induced by **K858**, while compound **41** presents roughly 50% of viable cells at 6.25 μM after 24 h exposure (Figure 3A,B); moreover, after 48 h compound **2** evidences only 20% of viable cells at 3.125 μM, while compound **41** presents only 30% of viable cells at 6.25 μM. Compound **30** shows a similar trend to compound **41**, exhibiting, after 24 h of treatment, at 6.25 µM 40% of viable cells (Figure 3C,D).

Compounds **2** and **30** differ from the **K858** structure only by slight differences on the C5 substituents, whereas compound **41** can be considered as a hybrid between the structure of **K858** and that of Filasenib and MK-0731 (other two well-established Eg5 inhibitors). Moreover, compound **41** displays a CLogP value (2.610), as an estimation of the lipophilicity of the compounds, more similar to **K858** (2.324) with respect to compound **30** (2.725) and more different from compound **2** (2.853). In order to better explore the chemical space within this scaffold and keeping in mind that the dose-response curves of the viability data for compounds **30** and **41** are comparable, we selected the two structurally different compounds **2** and **41** for further investigations establishing for compound **2** and **K858** 1 μM and for compound **41** 5 μM as selected concentrations. These doses were chosen because they are lower than IC_50_ and, therefore, can be considered subtoxic doses, suitable for further molecular investigations.

### 2.2. **K858**, Compounds **2** and **41** Selectively Inhibit Eg5 Resulting in Formation of Monopolar Spindle

**Figure 4:**
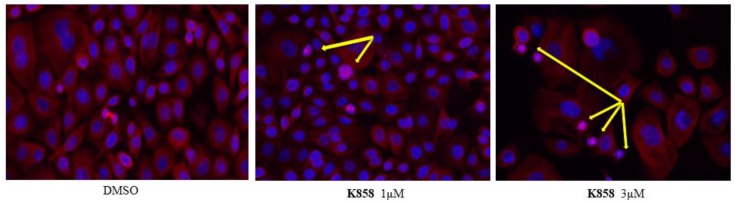
**β-tubulin immunofluorescence analysis in AGS cell line treated with K858 for 24 h**. AGS cells were treated for 24 h with 1 and 3 μM of **K858**. Tubulin cytoskeleton was visualized following fixation by immunostaining, DNA was stained with 4’,6-diamidino-2-phenylindole (DAPI). After 24 h of **K858** treatment the majority of AGS cells arrested in mitosis resulting in formation of monopolar spindle (yellow arrow).

**Figure 5:**
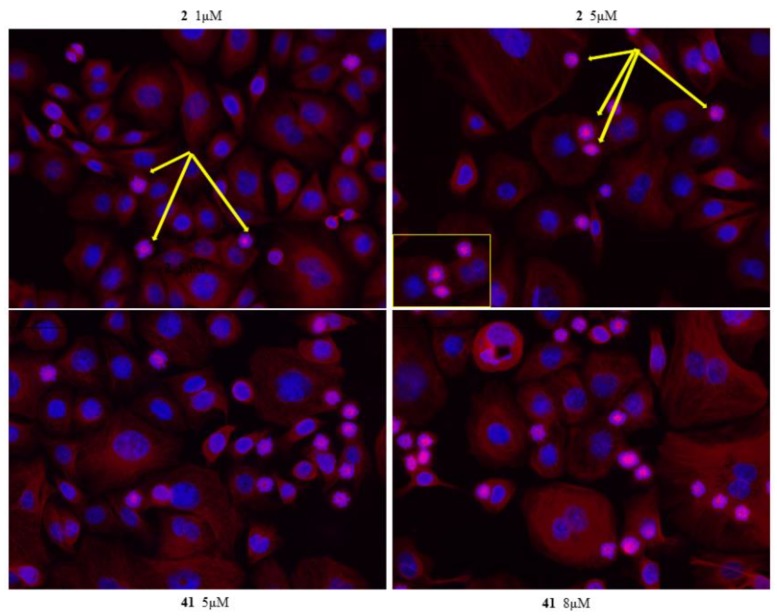
**β-tubulin immunofluorescence analysisin AGS cell line treated with compounds 2 and 41for 24 h.** AGS cells were treated for 24 h with 2 and 5 μM of compound **2** and with 5 and 8 μM of **41**. Tubulin cytoskeleton was visualized following fixation by immunostaining, DNA was stained with DAPI. After 24 h of compounds **2** and **41** treatment, the majority of AGS cells arrested in mitosis resulting in formation of monopolar spindle (yellow arrow).

To characterize mitotic slippage, we treated AGS with 1 and 5 μM of compound **2**, 5 and 8 μM of **41**, 1 and 3 μM of **K858** for 24 h. The morphology of microtubule cytoskeleton by immunostaining was evaluated (Figure 4 and Figure 5). Our data evidence that compounds **2**, **41** and **K858** prevent centrosome separation and promote the formation of monopolar spindle with a chromosomal alignment during mitosis at both concentration exposures in AGS cell line, indicating that a number of these cells are arrested in mitosis. Our results show that a treatment of 24 h with compounds **2**, **41** and **K858**, evidences different percentage of AGS cells with monoasters suggesting that the cells are sensitive to the different dose administrated. The number of monoasters is strongly increased when the cells are treated with compound **41** at 8 μM for 24 h compared to **K858**, compound **2** and DMSO. Furthermore, AGS cells exhibit a lower number of monoasters in cells treated with 1 μM of **K858** compared to cells treated with compound **2** at the same concentration for 24 h (Figure 4 and Figure 5).

### 2.3. Bax, Caspase-3, Nitric Oxide Synthase 2 (NOS-2), and Metalloproteinase 9 (MMP-9) Expression in Response to Kinesins Eg5 Inhibitors in AGS Cells

**Figure 6:**
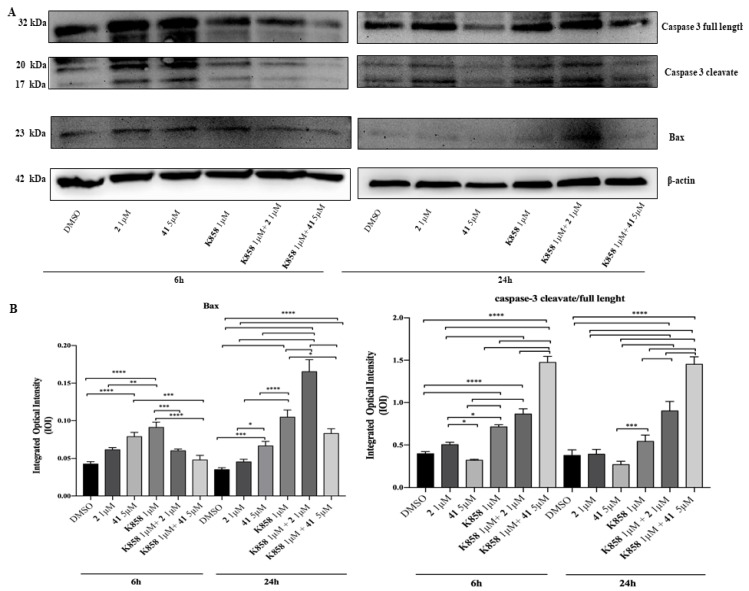
**Western blotting analysis of Bax and Caspase-3 expression in AGS cell line treated with compounds 2, 41, K858 as single agents and in co-treatment**. (**A**) Cells treated with DMSO (0.2%) were loaded as negative control. Each membrane was probed with β-actin antibody to verify loading consistency. Western blot is the most representative of three different experiments. (**B**) Histograms represent densitometric measurements of proteins bands expressed as integrated optical intensity (IOI) mean of three separate experiments. The error bars show standard deviation (± SD). Densitometric values analyzed by ANOVA (post hoc application of Tukey’s multiple comparison test) return significant differences. **** *p* < 0.0001, *** *p* < 0.0002, ** *p* < 0.0005, * *p* < 0.005.

To identify the effects of 1 μM of compounds **2**, **K858** and of 5 μM of compound **41** as single agents and in co-treatment with 1 μM of **K858** on the apoptotic process, Western blotting analysis of Bax and Caspase-3 was carried out after 6 and 24 h of compounds administration. After 6 and 24 h a significant increase in Bax expression is evidenced in cells exposed to compound **41** and **K858** as single agents compared to DMSO and to sample treated with compound **2**; after 24 h of exposure the co-treatment of compound **2** and **K858**, both used at 1 μM, reveals a strongly increase in Bax expression respect to DMSO and to all other experimental points. After 6 h of exposure a higher expression of the pro-apoptotic protein is reported in samples treated with 1 μM **K858** respect to samples treated with compound **2** as single agent and respect to compounds **2** and **41** in co-administration with **K858**. Moreover, after 24 h of treatment, a remarkable increase in protein level is shown in cells treated with compound **2** in co-administration with **K858** respect to all other experimental points (Figure 6).

In addition, after 6 and 24 h of exposure cleaved caspase-3/full length ratio has a similar trend at both time points; a remarkable increase in samples in co-treatment with compounds **2** and **41** with 1 μM **K858** compared to DMSO is detectable (Figure 6).

**Figure 7:**
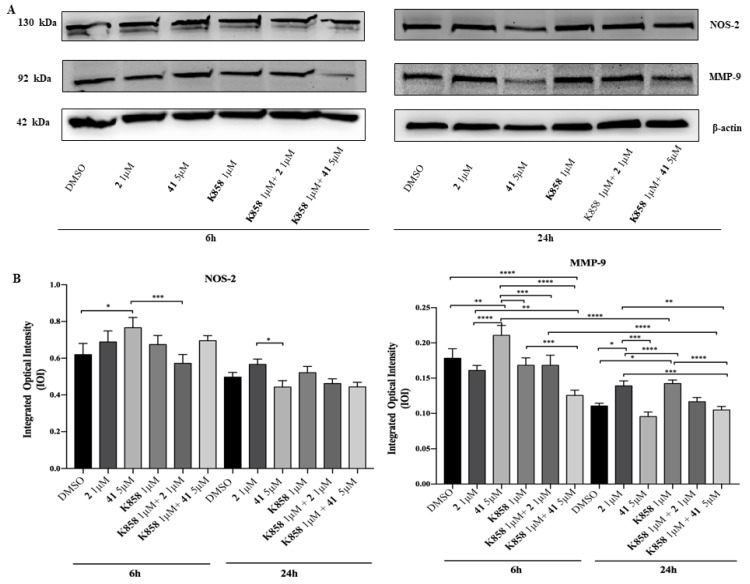
**Western blotting analysis of Matrix Metalloproteinase 9 (MMP-9) and Nitric Oxide Synthase (NOS-2) expression in AGS treated with compounds 2, 41, and K858 as single agents and in co-treatment**. (**A**) Cells treated with DMSO (0.2%) were loaded as negative control. Each membrane was probed with β-actin antibody to verify loading consistency. Western blot is the most representative of three different experiments. (**B**) Histograms represent densitometric measurements of proteins bands expressed as integrated optical intensity (IOI) mean of three separate experiments. The error bars show standard deviation (±SD). **** *p* < 0.0001, *** *p* < 0.0002, ** *p* < 0.0005, * *p* < 0.005.

Then, a Western blotting analysis of Nitric Oxide Synthase (NOS-2), involved in the inflammatory event induction, was carried out. After 6 h, NOS-2 expression is slightly augmented in samples exposed to 5 μM of compound **41** compared to DMSO sample and considerably augmented in respect to compound **2** in co-administration with 1 μM **K858**. Additionally, after 24 h a slight increase is found in cells treated with **2** in respect to cells treated with compound **41** (Figure 7). Moreover, a Western blotting of Matrix Metalloproteinase 9 (MMP-9), a protease responsible for the remodeling and turnover of extracellular matrix, was carried out. After 6 h, MMP-9 expression is remarkably increased in samples exposed to 5 μM of compound **41** compared to DMSO and to all other experimental points, while after 24 h cells treated with compound **41** show a significantly lower MMP-9 expression level than cells treated with compound **2** and **K858**. At both time points samples treated with compound **41** in co-administration with **K858** report a notable reduction of protein level expression compared to samples exposed to compound **2** as a single agent, while after 6 h no significant difference in samples treated with compound **2** in co-administration with **K858** with respect to DMSO and samples exposed to compounds **2** and **41** is evidenced. Conversely, after 24 h exposure a slight reduction in samples treated with compound **2** in co-administration with **K858** with respect to samples treated with compound **41** and **K858** is reported (Figure 7).

### 2.4. Effects of Novel Kinesin Eg5 Inhibitors and **K858** in Cell Migration

**Figure 8:**
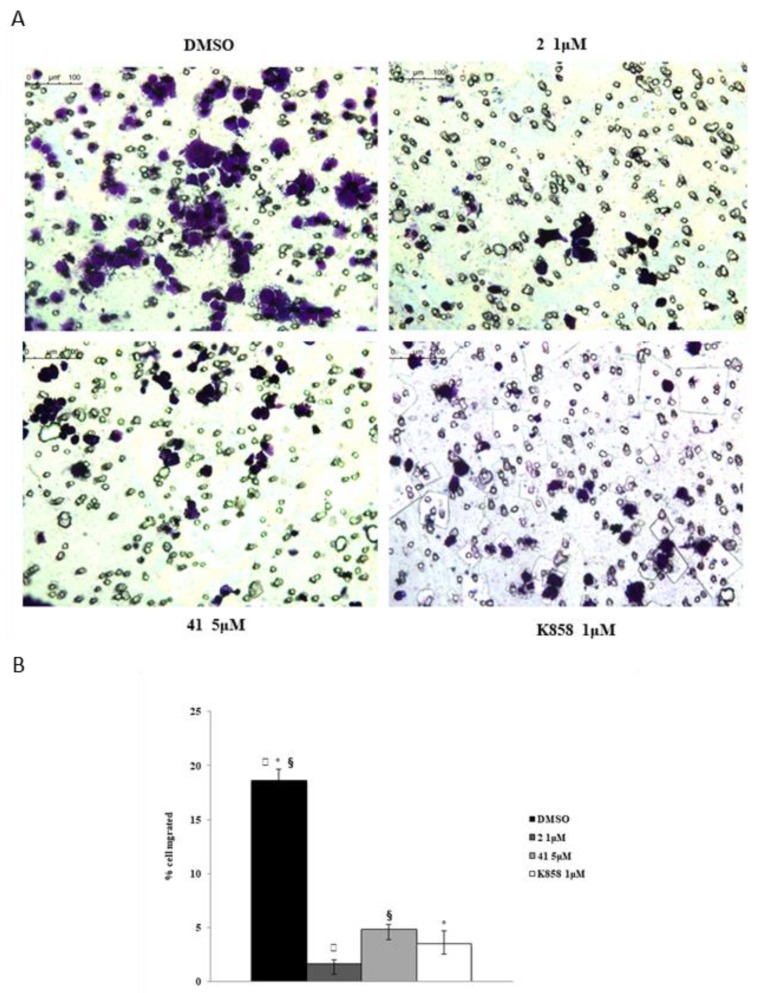
Transwell migration assay in AGS cell line in the presence of compounds **2**, **41** and **K858**. Dose treatments are 1 μM for compound **2** and **K858**, and 5 μM for compound **41**, respectively. (**A**) Images represent migrated cells after staining with crystal violet. (**B**) Histogram represents densitometric analysis determined by quantifying thresholded area for violet color in 10 fields for each of three slides per sample. Data are presented as mean ± standard deviation. *K858 1 μM vs. DMSO; ^§^compound **41** 5 μM vs. DMSO; ^▯^compound **2** 1 μM vs. DMSO: *p* < 0.0001.

A transwell migration assay was performed by means of an 8 μM pore size polycarbonate membrane in AGS cell line with or without compound **2** and **K858** at 1 μM and compound **41** at 5 μM. Cells were exposed for 24 h to medium in the absence or in presence of chemoattractant (FBS) with or without the compounds, and then migrated cells were colored with crystal violet. All Eg5 inhibitors induce a considerable reduction of cell migration in respect to DMSO experimental point (Figure 8).

## 3. Discussion

A large plethora of recent papers evidenced the importance of specific gene mutations or protein dysregulation, such as PI3K/Akt, F-box proteins, and NF-κB, involved in the cell cycle progression [16,17,18,19]. These targets were shown to be related to proliferation, invasion, angiogenesis, and metastasis of gastric cancer cells in vitro and in vivo, thus establishing a new scenario for the personalized therapy of this malignancy. The regulation of key signaling pathways and cell cycle in gastric cancer could be finely modulated by newly synthesized compounds and, currently, this approach resulted as one of the most useful strategies to develop innovative therapeutic protocols.

Several studies have reported that Kinesin Eg5, a motor protein involved in the assembly and maintenance of the bipolar spindle during mitosis, is highly expressed in different types of tumors and it is frequently associated with drug resistance [20,21]. Therefore, the development of more potent kinesin inhibitors for the clinical applications in cancer therapies is urgently needed as an alternative to drugs targeting the mitotic spindle, also designed to reduce the side effects of taxanes, such as neurotoxicity [2]. A recent study, published by Imai et al., demonstrated that *Eg5* mRNA expression was found increased in gastric adenocarcinoma respect to normal tissue [7]. These findings underlined that Eg5 plays an important role in gastric cancer and the development of novel Eg5 inhibitors could be a promising approach for cancer treatments. For this reason, the biological effects of novel kinesin Eg5 inhibitors, synthesized in the pharmaceutical chemistry laboratory of our Department, against human adenocarcinoma cell line, were evaluated.

Surprisingly, two novel Eg5 inhibitors tested, namely compounds **2** and **41**, evidenced a strong capability to significantly reduce adenocarcinoma cell viability, especially compound **2** which possesses an IC_50_ value lower than 3 μM, comparable with **K858** IC_50_ value. These findings lead us to keep these promising molecules in monotherapy and in combination with **K858**. As reported in the literature, Eg5 inhibition results in the formation of monoaster spindles which is thought to lead to mitotic catastrophe and apoptosis [22]. This aspect, which is dose-dependent, is clearly demonstrated in our experimental model where compounds **2**, **41** and **K858**, by inhibiting Eg5 protein, lead to the formation of several monopolar spindles [12]. Mitotic braking, due to an improper mitotic spindle formation, may result in the induction of apoptosis by activating several pro-apoptotic factors, among which Bax and Caspases are included [23].

The capability of kinesin inhibitors to induce apoptosis was firstly investigated by evaluating Bax pro-apoptotic factor. Compounds **41** and **K858** in monotherapy are able to trigger the apoptotic cascade by significantly increasing Bax expression, as widely reported for both first-generation (taxanes and vinca alkaloids) and novel drugs which all act disrupting spindle dynamics [23,24]. Secondly, cleaved caspase-3 expression, a well-known downstream mediator of the intrinsic apoptotic pathway, was measured, finding that compounds **2** and **41** only in co-administration with **K858** are able to progress the apoptotic pathway through caspases activation. It can be hypothesized that compounds **2** and **41** administered with **K858** result in a synergistic effect, enhanced by **K858**, resulting in the activation of the mitochondrial apoptotic pathway.

Since it is recognized that in several cancers the pro-tumoral effects are directly correlated with the NOS-2 activity [25], an aggressive cancer biomarker, its level expression was also investigated. At an early stage, compound **41** seems to upregulate NOS-2 expression, while, surprisingly both compounds **41** and **2** in co-administration with **K858** are able to downregulate the protein expression. These findings led us to assume that the reduction of the NOS-2 is largely due to a likely synergistic effect exhibited when novel compounds are in combination with **K858**.

The prognostic value of MMP-9 in different types of cancer has been clearly identified, also demonstrating that its expression promotion and its transformation to active MMP-9, is dependent on NO synthesis, performed by the NOS-2 enzyme [25,26,27]. This finding is confirmed in our model where MMP-9 expression trend is overlapping to NOS-2. In particular, this result evidences that, at an early time, only compound **41** in combination with **K858** is able to downregulate the MMP-9 expression thus probably reducing the invasiveness of cancer cells. However, this is in contrast to what was reported by the migration assay in which both novel compounds and **K858** in monotherapy markedly reduce cell migration. This could be explained admitting that an alternative pathway is recruited to contrast cell migration when compounds **2** and **41** are administered.

To conclude it can be argued that our novel Eg5 inhibitors in monotherapy are able to counteract gastric adenocarcinoma cells proliferation and invasiveness, moreover, the synergistic effect, enhanced by the combination therapy with **K858** could be a promising strategy to induce cell death through the apoptotic cascade activation. Taken together these results highlight the importance of understanding and exploring the biological effects of different novel Eg5 kinesin inhibitors administrated in monotherapy and in combination with **K858** as potential strategy to counteract gastric cancer.

## 4. Materials and Methods

### 4.1. Tested Compounds

**K858** and the other thiadiazoline analogues were synthesized, purified, and characterized as previously reported [28]. CLogP values were calculated by ChemBioDraw Ultra^®^ 12.0.

### 4.2. Cell Lines

AGS human gastric adenocarcinoma cell line (ECACC 89090402, Sigma Aldrich, Milan, Italy) was cultured in Ham’s F12 medium with 10% of foetal bovine serum (FBS), 1% of penicillin/streptomycin, and 1% of l-glutamine (all purchased by EuroClone, Milan, Italy). Cell culture was kept within an incubator in a humidified atmosphere with 5% CO_2_ at 37 °C.

### 4.3. MTT Assay

The cell density chosen for AGS cells, within a 96-well tissue culture plate, was of 10,000/well. The metabolic activity of AGS cells was measured after 24, 48, and 72 h of exposure to novel kinesin Eg5 inhibiting compounds **2**, **4**, **26**, **30**, **31**, **41,** and **44** at 0.5, 5, 50, 100 μM and with **K858** at doses ranging from 0.5, 5, and 50 μM by means of a MTT (3-(4,5-dimethylthiazol-2-yl)-2,5-diphenyltetrazolium bromide) test (Sigma Aldrich, Milan, Italy). The MTT test is based on the reduction of capability MTT, performed only by viable cells, into a violet formazan salt. DMSO was used to dissolve kinesin Eg5 inhibitors with a final DMSO concentration of 0.2%. At the established experimental times, the medium was replaced by a fresh with 0.5 mg/mL MTT and incubated with cells at 37 °C for 4 h. The plate was then probed in DMSO for 30 min at 37 °C to allow formazan salt dissolution. The colored solution was then read at 540 nm through a microplate reader (Multiskan GO, Thermo Scientific, Waltham, MA, USA). Data obtained without cells were established as background. Viability level was normalized with values obtained from cells treated with DMSO.

### 4.4. Immunofluorescence Analysis

Cells were seeded on glass Millicell EZ slides (Merck Millipore, Darmstadt, Germany), treated with compound **2** at 1 and 5 μM, **K858** at 1 and 3 μM, while compound **41** at 5 and 8 μM for 24 h. Cell monolayer was fixed for 15 min at 4 °C with paraformaldehyde 4% in PBS. After two rinses in PBS, the permeabilization of the cells was performed with Triton-X100 0.3% in PBS for 10 min at 37 °C. To detect β-tubulin, slides were then probed in 5% normal goat serum (NGS; Sigma Aldrich, St. Louis, MO, USA) in PBS for 30 min at room temperature. An incubation of 60 min in the presence of β-tubulin mouse monoclonal antibody (Santa Cruz Biotechnology, Santa Cruz, CA, USA), diluted 1:100 in PBS, 5% Tween 20, and 2% bovine serum albumin (BSA) at 37 °C in agitation was then carried out. Slides were rinsed with PBS and incubated for 45 min at 37 °C in agitation with tetramethylrodamine (TRITC) conjugated with goat anti-rabbit immunoglobulin (IgG) antibody (Sigma Aldrich, St. Louis, MO, USA), diluted 1:50 in PBS, 5% Tween 20, and 2% BSA. Nuclei were counterstained with 4’,6-diamidino-2-phenylindole (DAPI; Santa Cruz, CA, USA) and slides mounted with glycerol-1,4-diazabicyclo[2.2.2]octane (DABCO; Sigma Aldrich, St. Louis, MO, USA). The negative control was carried out by incubating the slides without the primary antibody. The slides were analyzed with a Leica DM 4000 microscope (Leica Cambridge Ltd, Cambridge, UK) equipped with Leica DFC 320 Videocamera (Leica Cambridge Ltd, Cambridge, UK) to obtain and examine computerized images.

### 4.5. Western Blot Analyses

The cell lysates (20 µg) underwent electrophoresis and were transferred to the nitrocellulose membrane. The latter were blocked in 5% of non-fat milk in PBS 0.1% Tween-20, then incubated in the presence of mouse monoclonal anti-β actin antibody (antibody dilution 1:5000) (A5316 Sigma, St. Loius, MO, USA), rabbit polyclonal anti-Nitric Oxide Synthase-2 (NOS-2) antibodies (antibody dilution 1:200) (sc-651 purchased by Santa Cruz biotechnology, Santa Cruz, CA, USA), mouse monoclonal anti-Matrix Metalloproteinases-9 (MMP-9), and anti-Bax antibodies (antibodies dilution 1:200) (sc-5302 and sc-7480, respectively, both purchased by Santa Cruz biotechnology, Santa Cruz, CA, USA), goat polyclonal anti-caspase-3 antibody (antibody dilution 1:200) (sc-1225 purchased by Santa Cruz biotechnology, Santa Cruz, CA, USA). Samples were then probed with specific enzyme conjugated IgG horseradish peroxidase. Immunoreactive bands were revealed by ECL system (Amersham Int., Buckunghamshire, UK) and underwent densitometric analysis. Values obtained from densitometry, expressed as Integrated Optical Intensity (IOI), were evaluated with a CHEMIDOC XRS system through the QuantiOne 1-D analysis software (BIORAD, Richmond, CA, USA). Data were normalized with densitometric values derived from β-actin loading control.

### 4.6. Transwell Migration Assay

AGS cell migration was evaluated through a 24-well Transwell Boyden chamber consisting of 8 µm pore size membranes (Corning, Lowell, MA, USA). Suspended AGS human gastric adenocarcinoma cells were separately treated with compound **2** and **K858** at 1 µM and **41** at 5 µM and in serum-free Ham’s F12 at cell density 50,000/150 μL, and then added to the upper chamber of an 8 μm pore size insert. Ham’s F12 with 10% FBS was added to the lower chamber as a chemoattractant allowing it to migrate towards a medium containing 10% FBS located in the lower chamber. AGS were left for 24 h at 37 °C within the incubator, the non-migrating cells on the upper chamber were then taken away with a cotton swab, while the cells migrated to the lower surface of the membrane were colored with crystal violet for 10 min at room temperature. Images were acquired with a light microscope equipped with a Leica DFC 320 camera (magnification 20 ×). Images were examined with Leica Application Suite-X (LAS-X) analysis software. Considering that cells stained by crystal violet represent the migrated cells, the migration level was established by measuring the surface covered by colored cells, as already reported elsewhere [29].

### 4.7. Statistics

Inhibitory Concentration of 50% cell population (IC_50_) values was calculated by Prism 7 (GraphPad). Statistical significance was established with GraphPad 7 software by means of *t*-test and Ordinary One-Way ANOVA followed by post-hoc Tukey’s multiple comparisons tests. Values of *p* < 0.05 were considered statistically significant.
